# Phenotypic plasticity of growth ring traits in *Pinus hartwegii* at the ends of its elevational gradient

**DOI:** 10.3389/fpls.2023.1072638

**Published:** 2023-09-20

**Authors:** Lizbeth Carrillo-Arizmendi, J. Jesús Vargas-Hernández, Philippe Rozenberg, Marlin Pérez-Suárez, Angel Roberto Martínez-Campos

**Affiliations:** ^1^ Instituto de Ciencias Agropecuarias y Rurales, Universidad Autónoma del Estado de México, Estado de México, Mexico; ^2^ Department of Forestry Sciences, Colegio de Postgraduados, Montecillo, Texcoco, Estado de México, Mexico; ^3^ UMR 0588 BIOFORA, INRAE Val de Loire, Cedex, Orleans, France

**Keywords:** radial growth, reaction norms, climate, Nevado de Toluca, La Malinche

## Abstract

**Introduction:**

Phenotypic plasticity (PP) could be an important short-term mechanism to modify physiological and morphological traits in response to climate change and global warming, particularly for high-mountain tree species. The objective was to evaluate PP response of growth ring traits to temperature and precipitation in *Pinus hartwegii* Lindl. populations located at the ends of its elevational gradient on two volcanic mountains in central Mexico (La Malinche and Nevado de Toluca).

**Methods:**

Increment cores collected from 274 P*. hartwegii* trees were used to estimate their PP through reaction norms (RN), which relate the ring width and density traits with climate variables (temperature and precipitation). We estimated the trees’ sensitivity (significant RN) to climatic variables, as well as the relative proportion of RN with positive and negative slope. We also estimated the relationship between the PP of ring width and density traits using correlation and Principal Component (PC) analyses.

**Results:**

Over 70% of all trees showed significant RN to growing season and winter temperatures for at least one growth ring trait, with a similar proportion of significant RN at both ends of the gradient on both mountains. Ring width traits had mostly negative RN, while ring density traits tended to have positive RN. Frequency of negative RN decreased from lower to higher elevation for most traits. Average PP was higher at the lower end of the gradient, especially on LM, both for ring width and ring density traits, although high intrapopulation variation in PP was found on both mountains.

**Discussion:**

Results indicate that *P. hartwegii* presents spatially differentiated plastic responses in width and density components of radial growth. PP was particularly strong at the lower elevation, which has higher temperature and water stress conditions, putting these populations at risk from the continuing global warming driven by climate change.

## 1 Introduction

Over the past decades, ambient temperature has increased (IPCC, 2021) and levels and patterns of precipitation have changed. As a result, it is estimated that heat waves (periods of abnormally hot temperatures with a duration of days or weeks) and droughts are becoming more intense at a global level, with important effects on terrestrial ecosystems (Feeley et al., 2020). Organisms must adjust the mechanisms of their physiological (e.g., carbon fixation, development of new tissue, etc.) and ecological processes (regeneration, facilitation, etc.) through adaptation and/or acclimatization to these changing conditions, migrate, or face the risk of extinction (Futuyma and Moreno, 1988). Migration and the development of new adaptive mechanisms are relatively slow, long-term processes; therefore, acclimatization mechanisms, which occur in the short term, are especially relevant in this scenario (Matesanz et al., 2010).

Acclimatization mechanisms involve fine changes to an organism’s development, morphology, and physiology. In plants, this can lead to increased tolerance to abiotic stress (Matesanz et al., 2010) and with it, an increase in survival and persistence under new climate scenarios. These mechanisms are manifested through phenotypic plasticity (PP), the capacity of a genotype to present different phenotypes in response to changes in its environment (Bradshaw, 1965; DeWitt and Scheiner, 2004). There are different approaches and methods to estimate the degree of PP in specific functional traits of interest (Sultán, 2003; Valladares et al., 2006; Escobar-Sandoval et al., 2021). One of these approaches is the estimation of reaction norms (RN), which quantify PP as the regression of an individual’s phenotypic trait in response to changes in a given environmental variable (Valladares et al., 2006; Sultán, 2007; Arnold et al., 2019). Thus, RN is a graphical model (linear or curvilinear regression) that shows the genotype × environment interaction and indicates (through its slope or curve) the degree of PP (Valladares et al., 2006).

High mountain forest ecosystems are especially vulnerable to climate change due to their harsh abiotic conditions, including poorly developed soil, high UV radiation, presence of snow, and low temperatures (Körner, 2007). As such, PP plays a particularly important role in the development and survival of sessile and long-lived organisms, like high-mountain tree species, facing adverse environmental conditions (Bradshaw, 1965; Silvestre et al., 2012; Santini et al., 2018). Given their long lifespan, trees confront innumerable climate fluctuations with distinct frequency and intensity (Rehfeldt et al., 2001), making them more likely to encounter extreme climate events which are reflected through the modification of their functional traits. Furthermore, these modifications are physically recorded as growth rings. Radial growth is one of the best indicators of PP, since both the width and the density of the tissue formed during xylogenesis are particularly sensitive to fluctuations in ambient temperature and rainfall (Engelbrecht, 2012; Meng et al., 2020) and can be quantified by measuring the growth rings. Plant species that are found growing at the limits of their elevational distribution generally present more plastic responses in their functional traits as a survival mechanism (Sultán, 1987), given the variability in temperature, levels, and patterns of rainfall, and their interaction with elevation. It is thus expected that radial growth of trees will be impacted differently depending on their position along an elevational gradient (Fritts, 1976; Körner, 2007; Carrillo-Arizmendi et al., 2022a).

The tree species *Pinus hartwegii* has attracted particular interest in the context of climate change (Pérez-Suárez et al., 2021), due to its wide altitudinal distribution range (3,000 – 4,000 m). Furthermore, it is the tree species with the highest recorded elevational distribution, with records at 4,200 m. Therefore, *P. hartwegii* has developed great resistance to the adverse conditions for arboreal growth (poor soils, extreme low temperatures, short growing seasons, etc.), present at high elevations in Mexico, Guatemala, and Honduras (Neuner, 2014; Alfaro-Ramírez et al., 2017; Manzanilla-Quiñones et al., 2019). Since its biological and ecological processes are adapted to extreme low temperatures, *P. hartwegii* has been pointed out as a particularly vulnerable tree species to global warming. Under projected climate trends, the distribution area suitable for *P. hartwegii* could be reduced by 30-70% in the next 50 years (Alfaro-Ramírez et al., 2020). This will undoubtedly have important consequences in many different contexts and spatial scales. On one hand, *P. hartwegii* forests have high value in the regulation of local and regional climate, in addition to other important ecological services. Furthermore, this species is used for construction and furniture making, with strong impact on the local and regional economies (Sánchez, 2008; Torres-Rojo, 2015; Moctezuma-López and Flores, 2020). Therefore, understanding the potential impact of global warming on the radial growth of *P. hartwegii* and the role of PP to adjust the growth response of trees would allow the establishment of efficient preservation and management strategies.

In the present study, we therefore aimed to determine the degree of PP of growth ring traits in *P. hartwegii* trees under harsh environmental conditions at the ends of its elevational distribution on two mountains in central Mexico — “La Malinche” (LM) and “Nevado de Toluca” (NT). For this, we proposed the hypothesis that the PP will show differential adaptive mechanisms between growth ring traits (i.e., width and density components) in response to interannual fluctuations in temperature and rainfall at the extremes of the elevational gradient, with compensatory effects at the population level, but not necessarily at the individual level. Our specific objectives were to: (1) evaluate PP in growth ring traits of *P. hartwegii* trees in response to interannual climate fluctuation; (2) determine the potential interrelationships between the PP of width and density traits in the growth rings; and (3) evaluate the effect of elevation and mountain on the expression of PP in growth ring traits to the climatic factors.

## 2 Materials and methods

### 2.1 Study sites

**Figure 1:**
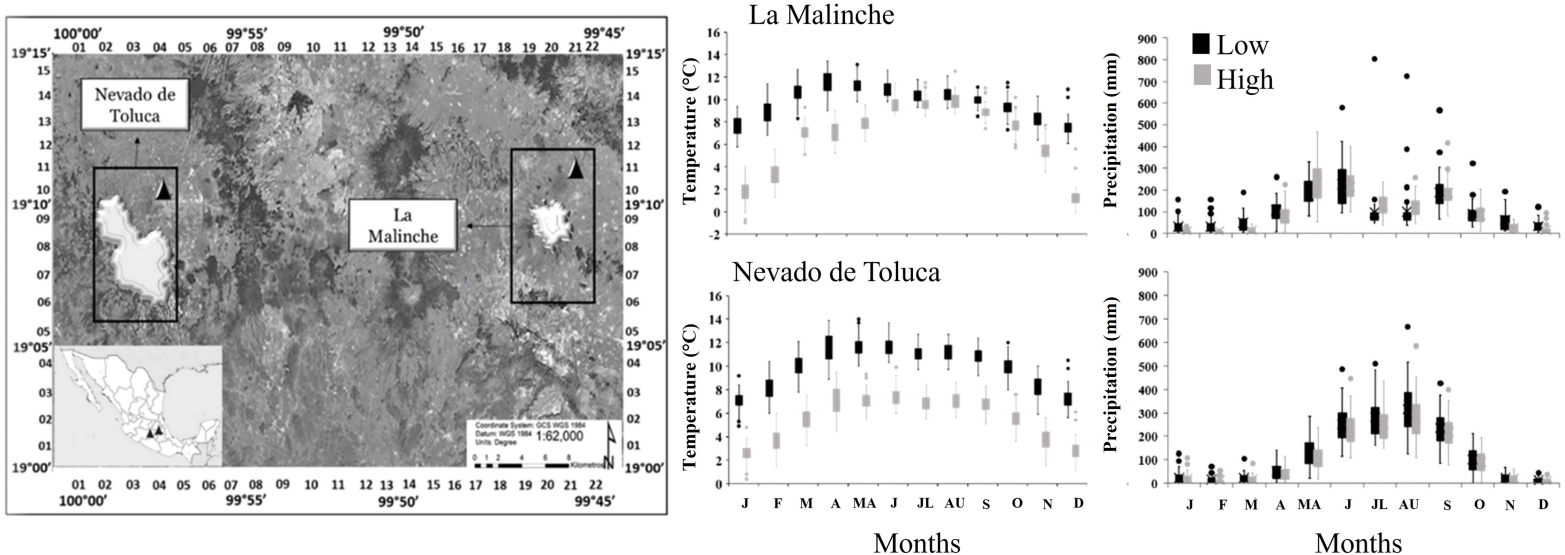
Left: Geographical location of the study area including the two mountain sites, Nevado de Toluca and La Malinche. Right: Climate diagrams with monthly mean temperature and precipitation values at the geographic coordinates sampled at the lower (black) and upper (gray) ends of the elevation gradient on the two mountains. Data were extracted from the ClimateNA database for the 1965-2016 period.

This study was carried out on two mountains in central Mexico: “La Malinche” (LM) and “Nevado de Toluca” (NT) (**Figure 1**). LM is located between the states of Tlaxcala and Puebla and has a maximum elevation of 4,461 m (Acosta-Pérez and Kong, 1991). NT is located in Mexico State, between the municipalities of Toluca and Tenango del Valle, and has a maximum elevation of 4,680 m (Körner and Paulsen, 2004). Both mountains belong to the Trans-Mexican Volcanic Belt, however, there is a distance between the mountains of 181.6 km. On both mountains there are widely different conditions between the lower and upper extremes of the elevational gradient. The data from the model ClimateNA (Wang et al., 2016) indicate that the mean annual temperature (MAT) of LM is 9.7°C at the lower end of the elevation gradient (**Table 1**) and 6.6°C at the upper end. The mean annual precipitation (MAP) at the lower end of LM it is 1208.2 mm and at the upper end is 1132.1 mm. On NT, the MAT is 9.9°C at the lower end and 5.5°C at the upper, and MAP is 1458.3 mm at the lower end and 1299.1 mm at the upper (**Figure 1**). In addition to *P. hartwegii*, which is the species with the highest elevation distribution range (3500 - 4200), other tree species can be found on both mountains, such as *P. montezumae* Lamb. (3,000 - 3,200 m) and *Abies religiosa* Kunth Schltdl. & Cham. (2,800 - 3,400 m); as well as grasslands, alpine zacatonal, and highland paramo (4,000 - 4,400 m) (CONANP, 2013a; CONANP, 2013b).

**Table 1:** Geographic coordinates, number, and age of the trees sampled per site at the extremes of the elevation gradient on La Malinche (LM) and Nevado de Toluca (NT) mountain.

Mountain	Elevation end	Elevation (m)	Latitude (N)	Longitude (O)	Samples (No. Trees)	Average age (age range) in years
**LM**	**Lower**	3,472	19°15.509’	98°1.7050’	25	38 (64-24)
		3,444	19°15.560’	98°1.8550’	25	30 (24-62)
		3,363	19°15.785’	98°12.138’	25	68 (35-100)
**LM**	**Upper**	4,100	19°24.091’	98°03.370’	25	49 (33-59)
		3,966	19°14.474’	98°1.9980’	25	78 (34-158)
		3,943	19°14.432’	98°1.8920’	25	34 (80-18)
**NT**	**Lower**	3,400	19°7.3630’	99°46.335’	33	82 (16-104)
		3,379	19°10.461’	99°48.754’	31	85 (63-139)
**NT**	**Upper**	4,126	19°7.2610’	99°45.229’	30	79 (37-169)
		4,000	19°9.4520’	99°48.449’	30	79 (33-147)

### 2.2 Sampling design and sample processing

On each mountain, sampling plots were selected at the lower and upper limits of the elevational distribution gradient of *P. hartwegii*. Three sites were sampled at each elevation on LM and two per elevation on NT (**Table 1**). The sites were selected based on the criteria of being low competition stands of mature *P. hartwegii* trees, without evidence of disturbance due to logging, wildfire, or pests. At each site, we selected between 25 and 31 dominant adult trees with a straight trunk, over 40 cm in diameter at breast height. For each selected tree, we extracted a wood core from the bark to the center of the trunk using a 5-mm diameter increment borer (HAGLOF ^®^, Suiza) at 1.30 m height above the ground. We sampled a total of 274 trees.

The collected increment cores were air-dried at room temperature and longitudinally sawn to obtain 1.57 mm thick transverse sections. Then, the resin was extracted with a bath in a water-pentane solution (2:1) for 48 hours. Each sample was exposed to a source of X-rays for 25 min in the wood densitometry laboratory of the Institut National de Recherche sur L’agriculture, L’alimentation et L’environnement, INRAe Val de Loire Center, Orleans, France. This was based on the procedure developed by Polge (1978) and described by Mothe et al. (1998). Then, microdensity profiles were obtained by scanning the X-ray images at a resolution of 4,000 dpi with the software WinDendro, Regent Instruments Inc. (Guay et al., 1992). This software converts the gray levels of the pixels detected in the X-rays image to density values, calibrated with a standard of known physical and optical density (Schweingruber, 1996).

### 2.3 Annual tree-growth ring traits and detrending

The microdensity profiles obtained from the growth cores were imported into R (“R Development Core Team”, 2018). Then, we calculated the following traits related to the width and density of the growth rings: overall ring width (RW: mm), earlywood ring width (EWW: mm) latewood ring width (LWW: mm), overall mean ring density (RD: g cm^-3^), earlywood ring density (EWD: g cm^-3^), latewood ring density (LWD: g cm^-3^), maximum density (MAXD: g cm^-3^) and minimum density (MIND: g cm^-3^). This was done using R functions designed to obtain these variables (Rozenberg et al., 2002).

**Figure 2:**
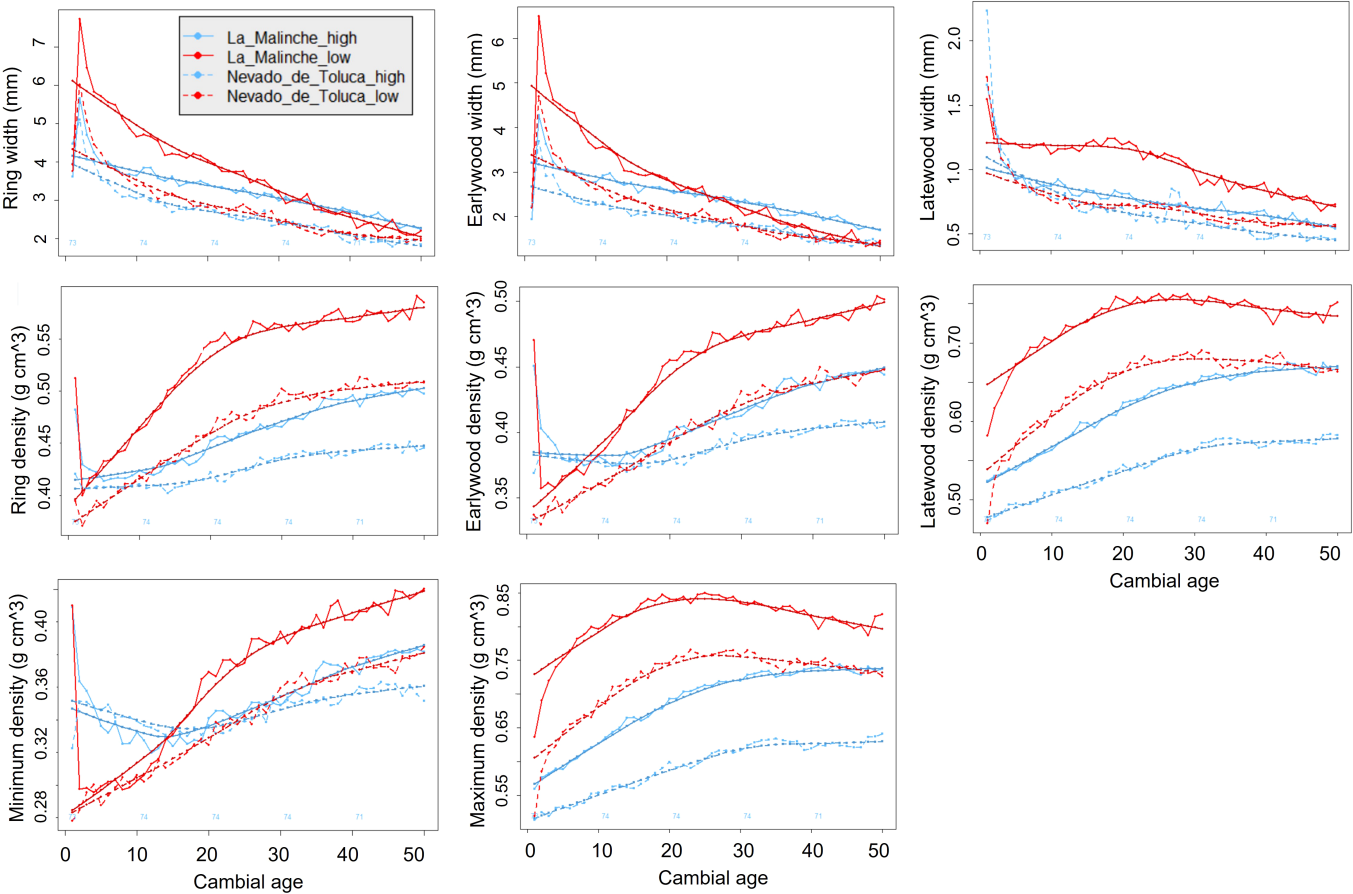
Fit of cambial age to the raw data of the time series for all width and density traits of growth rings of *P. hartwegii* trees located at the upper and lower ends of their elevation gradient on two mountains in central Mexico, “La Malinche” and “Nevado de Toluca”.

Cambial age influences width of growth rings and in general the properties of wood, especially during the juvenile stage (the first 10 to 25 rings). Although it is a natural effect of tree growth, this effect can introduce bias in the subsequent statistical analyses. We therefore removed the effect of cambial age by adjusting the raw data using the regional curve standardization (RCS) procedure, in the four elevation plots, as described below (**Figure 2**) (Esper et al., 2003).

Following Esper et al. (2003) and Rozenberg et al. (2020), we compared three methods to eliminate the effect of cambial age: raw data (no adjustment), residual RCS, and RCS ratio. Of these, the residual methods had the best fit to eliminate the effect of cambial age; we obtained one age-related curve for the lower end and another for the upper end for each of the mountains (LM and NT) (**Figure 2**). In each of the populations, we calculated the expressed population signal (EPS) statistic, which serves to determine the statistical quality of the chronology. The EPS value has a range of 0 to 1.0, where a value of 0.85 is considered acceptable (Buras, 2017). In all of our study sites, the EPS for RW was 0.88 or higher, above the reference point used to evaluate the quality of the chronologies. The residual chronologies and the time-dependent RCS ratios were obtained from the means of individual series. For the above analyses, we used the dendrochronology library for R, dplR (Bunn, 2008).

### 2.4 Climate variables

The climate variables for each sampling location were obtained with the software ClimateNA model v6.30 (Wang et al., 2016), which includes monthly data as well as annual mean (MAT), maximum (T_MAX_) and minimum (T_MIN_) temperature and total annual precipitation (TAP) for the 1964-2016 period. We used the monthly temperature data to calculate the mean temperature of the growing season (T_GS_; months of April through September), spring mean temperature (Tspring; months of April through June), and winter mean temperature (Twinter; months of January through March), The annual aridity index (AR_I_) was calculated with the equation AR_I_ = TAP/(MAT+10) (Fniguire et al., 2014). To verify that the data from the ClimateNA software accurately reflected the conditions at the study locations, we calculated the correlation between the ClimateNA data, and the data recorded by local meteorological stations. The meteorological stations were “Amaxac de Guerrero” (No. 29042; 3,320 m of elevation) and “Acxotla del Monte” (No. 29161; at 2,443 m of elevation) for LM and “Tenango” (No. 15122; at 2,858 m) and “Nevado de Toluca” (No. 15062; at 4,283 m) meteorological stations for NT. In all cases, there were very strong correlations between MAT and TAP values (r > 0.97), except in the case of TAP with meteorological station No. 29161, which had a moderate correlation (r = 0.59).

### 2.5 Statistical analysis

All the statistical analyses were done using the adjusted data for the growth ring traits for the 1964–2016 period. To reduce the number of variables to analyze (RW, EWW, LWW, RD, EWD, LWD, MAXD, MIND), we estimate the Pearson correlation coefficients among the growth ring traits, using the mean values per sampling site per year (showed in annexes). Pairs of variables that were strongly correlated (r > 0.9) were consolidated by selecting only one representative variable; this resulted in the selection of five variables for subsequent analyses: RW, LWW, RD, EWD and MAXD.

The RN for each ring trait to each climate variable was calculated for each individual tree based on the linear model Y_i_ = β_1_ + β_2_X_i_ + ϵ_ij_, where Y_i_ is the phenotypic trait (ring trait); X_i_ is the climate variable, β_1_ is the intercept, β_2_ is the slope (the PP value), and ϵ_ij_ is the model residuals. The model was fit using the lm function of R, from the R stats package (R Core Team, 2018). For each RN, we obtained the probability value (p) as well as the adjusted R^2^ value as an indicator of the goodness of fit of the linear model. When p < 0.05 we considered that the slope of the RN was significantly different from zero, demonstrating that the ring trait showed PP, and the regression coefficient (β_2_) was a quantitative estimate of the amount of PP of the ring trait to the climate variable for the corresponding tree. For the following statistical analyses, we only worked with the climatic variables: “T_GS_” and “Twinter”, because they were the ones with which *P. hartwegii* showed greater PP through the traits of its growth rings. It should also be noted that a previous research (Carrillo-Arizmendi et al., 2022b) carried out correlations between these two climatic variables indicating that there is a relatively weak relationship (0.460 ≤ |r| ≤ 0.619), such that these two variables are not providing redundant information, so we consider it is appropriate to include both in order to compare the plastic response of trees to these two climate variables and determine which one might be more reliable across populations. The relative frequency of trees with significant RN (RN_S_) for each growth ring trait-climate variable was calculated for each mountain/elevation combination (hereafter, “population”), to compare the overall “sensitivity” in PP of ring traits to different climate variables at the lower and upper end of the elevation gradient on both mountains. In addition, to identify possible relationships and/or compensatory effects on PP of growth ring traits, a correlation coefficient analysis and a principal component analysis (PCA) of the PP values for the ring traits were carried out. We also calculated the proportion of trees with positive and negative slope of RN_S_ for each ring trait. With these data, we determined whether there were differences in the proportion of trees with positive and negative RN_S_ between the two ends of the elevation gradient (lower and upper) and between mountains (LM and NT). The Glimmix procedure in SAS (SAS 9.4, 2019), with the link function for binary variables (proportions) was used for this purpose. A two-way ANOVA (SAS 9.4) was also done to evaluate the effect of elevation, mountain, and their interaction on the average slope value (β_2_) of the positive and negative RN_S_ for each ring trait and climate variable to determine whether the populations differed in the magnitude of PP.

## 3 Results

### 3.1 PP in growth ring traits of *P. hartwegii* trees in response to interannual climate fluctuation

**Figure 3:**
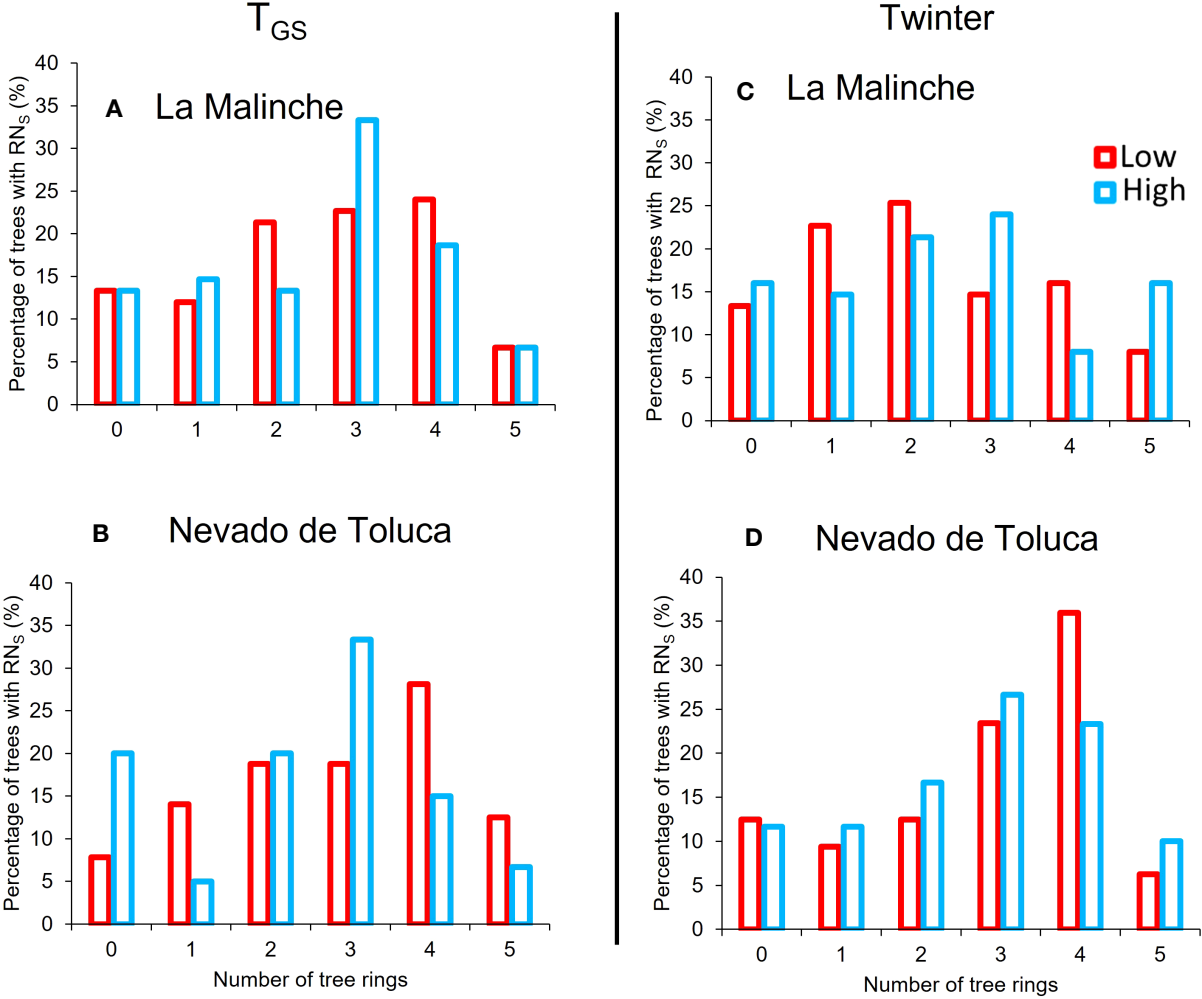
Relative frequency of *P. hartwegii* trees with significant reaction norms (RN) for one or more growth ring traits at each elevational extreme (lower: red; upper: blue) on each mountain; La Malinche (LM; **A**, **C**) and Nevado de Toluca (NT; **B**, **D**) in response to the growing-season temperature (T_GS_; **A**, **B**) and winter temperature (Twinter; **C**, **D**). The number 0 represents the proportion of trees with no significant RN for any growth ring trait; the other numbers represent the proportion of trees with significant RN for 1, 2, 3, 4 or all 5 growth ring traits. All bars add up to 100% in each series.

Growth ring traits were more “sensitive” to the climate variables T_GS_ and Twinter; that is, they had the overall largest number of significant RN with these two climate variables (**Table 2**) and a high percentage of trees with significant RN for more than one ring trait (**Figure 3**). Overall, the percentage of trees with significant RN for a growth ring trait was around 50% for both T_GS_ and Twinter across elevations and mountains, except for the RN related with Twinter at low elevation in LM mountain, were it was only 44.3% (**Table 2**). None of the other climate variables had similar percentages of significant RN across all populations. For instance, there was a high percentage of significant RN with mean annual temperature (MAT) in three populations but not at high elevation in LM. T_MAX_ was also important in three populations, but not at low elevation in LM and TAP was important only at the upper end in NT mountain. Given that T_GS_ and Twinter had the highest proportion of trees with significant RN, the remaining analyses were done only for these two climate variables. Looking at individual growth ring traits, the proportion of trees with significant RN to T_GS_ and Twinter differed only for RW, between mountains at the lower end, with a lower percentage on LM mountain (**Table 2**). Despite these differences among populations, the overall proportion of trees with significant RN in response to T_GS_ and Twinter was high on both mountains; over 70% of trees had significant RN for at least one growth ring trait, and over 40% of trees in all populations showed significant RN for three or more growth ring traits (**Figure 3**).

**Table 2:** Percentage of trees with significant reaction norms (RN_S_) for growth ring traits in relation to different climate variables at the two ends of the elevation gradient of *Pinus hartwegii* on two mountains in central Mexico.

Lower end							Higher end					
	RW	LWW	RD	EWD	MAXD	Overall	RW	LWW	RD	EWD	MAXD	Overall
La Malinche												
**TAP**	42.7	32.0	41.3	41.3	25.3	36.5	6.7	8.0	13.3	9.3	9.3	9.3
**MAT**	49.3	46.7	53.3	46.7	48.0	48.8	32.0	28.0	38.7	41.3	41.3	36.3
**T_GS_**	**42.7** ^X^	**38.7**	**64.0**	**57.3**	**49.3**	**50.4**	**53.3**	**44.0**	**50.7**	**52.0**	**49.3**	**49.9**
**Tspring**	30.7	26.7	42.7	44.0	30.7	34.9	46.7	40.0	42.7	42.7	41.3	42.7
**Twinter**	**49.3** ^X^	**37.3**	**45.3**	**44.0**	**46.7**	**44.3**	**54.7**	**46.7**	**49.3**	**48.0**	**42.7**	**48.2**
**T_MIN_**	20.0	18.7	16.0	18.7	18.7	18.4	8.0	8.0	8.0	12.0	4.0	8.0
**T_MAX_**	29.3	32.0	40.0	38.7	37.3	35.5	60.0	45.3	57.3	56.0	48.0	53.3
**AR_I_**	13.3	9.3	14.7	20.0	8.0	13.1	2.7	2.7	8.0	2.7	2.7	3.8
Nevado de Toluca												
**TAP**	25.0	15.6	26.6	26.6	9.4	20.6	66.7	58.3	56.7	53.3	48.3	56.7
**MAT**	76.6	59.4	53.1	57.8	60.9	61.6	65.0	56.7	55.0	51.7	41.7	54.0
**T_GS_**	**69.8** ^X^	**57.8**	**48.4**	**54.7**	**53.1**	**56.6**	**58.3**	**53.3**	**45.0**	**41.7**	**40.0**	**47.7**
**Tspring**	45.3	32.8	25.0	37.5	42.2	36.6	35.0	41.7	23.3	25.0	35.0	32.0
**Twinter**	**73.4** ^X^	**54.7**	**42.2**	**56.3**	**53.1**	**55.9**	**60.0**	**51.7**	**50.0**	**58.3**	**48.3**	**53.7**
**T_MIN_**	71.9	54.7	45.3	51.6	53.1	55.3	58.3	50.0	35.0	45.0	45.0	46.7
**T_MAX_**	64.1	53.1	45.3	50.0	51.6	52.8	66.7	58.3	51.7	56.7	51.7	57.0
**AR_I_**	0.0	4.7	3.1	1.6	3.1	2.5	10.0	3.3	0.0	0.0	5.0	3.7

RW, Ring width; LWW, Latewood width; RD, Overall ring density; EWD, Earlywood density; MAXD, Maximum density; TAP, Total annual precipitation (mm); MAT, Mean annual temperature (°C); T_GS_, Growing season temperature (°C); Tspring, Spring mean temperature (°C); Twinter, Winter temperature (°C); T_MIN_, Mean minimum temperature (°C); T_MAX_, Mean maximum temperature (°C); AR_I_, Aridity index. ^x^ Differences between mountain; P = 0.05.

### 3.2 Relationships between the PP of growth ring traits

**Figure 4:**
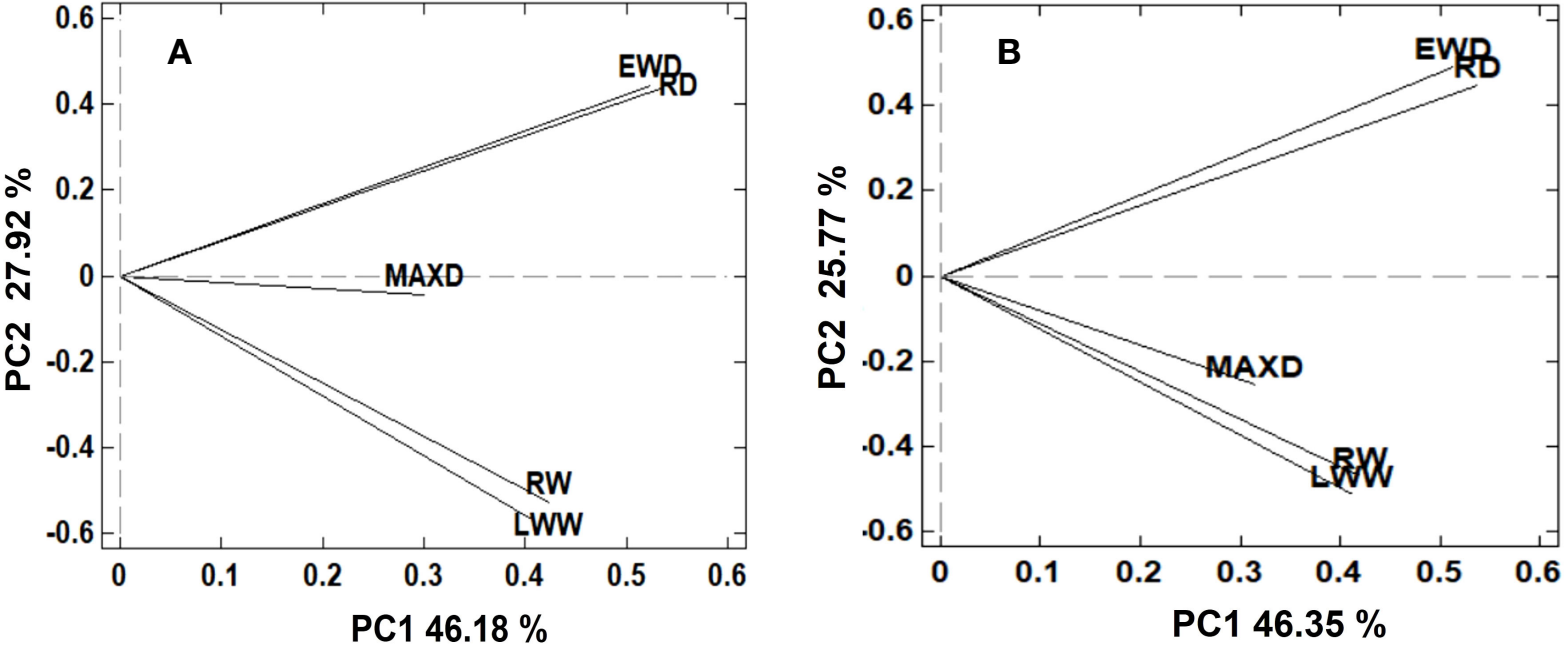
By-plot of the first two principal components (PC_1_ and PC_2_) obtained from the PC analysis of phenotypic plasticity (PP) values for growth ring traits (RW, LWW, RD, EWD and MAXD) associated with growing season temperature **(A)** and winter temperature **(B)**.

The correlation matrix and the first two PC obtained from the PCA showed that for both climatic variables (T_GS_ and Twinter), the PP of growth ring traits was separated in three distinctive groups: ring and latewood width in one group, ring and earlywood density in other, and maximum density in the third group (**Figure 4**). In the correlation matrix of PP values associated with both T_GS_ and Twinter, RW and LWW were strongly correlated, as well as RD and EWD.In general, the PP of growth ring traits in response to the two climate variables (T_GS_ and Twinter) showed a similar structure. Thus, the PP of each ring variable in response to T_GS_ was strongly correlated (r > 0.87) with the PP of the same variables in response to Twinter. The exception was LWW, where the correlation was slightly weaker (r = 0.76).

### 3.3 Effect of elevation and mountain on the expression of PP in growth ring traits

**Figure 5:**
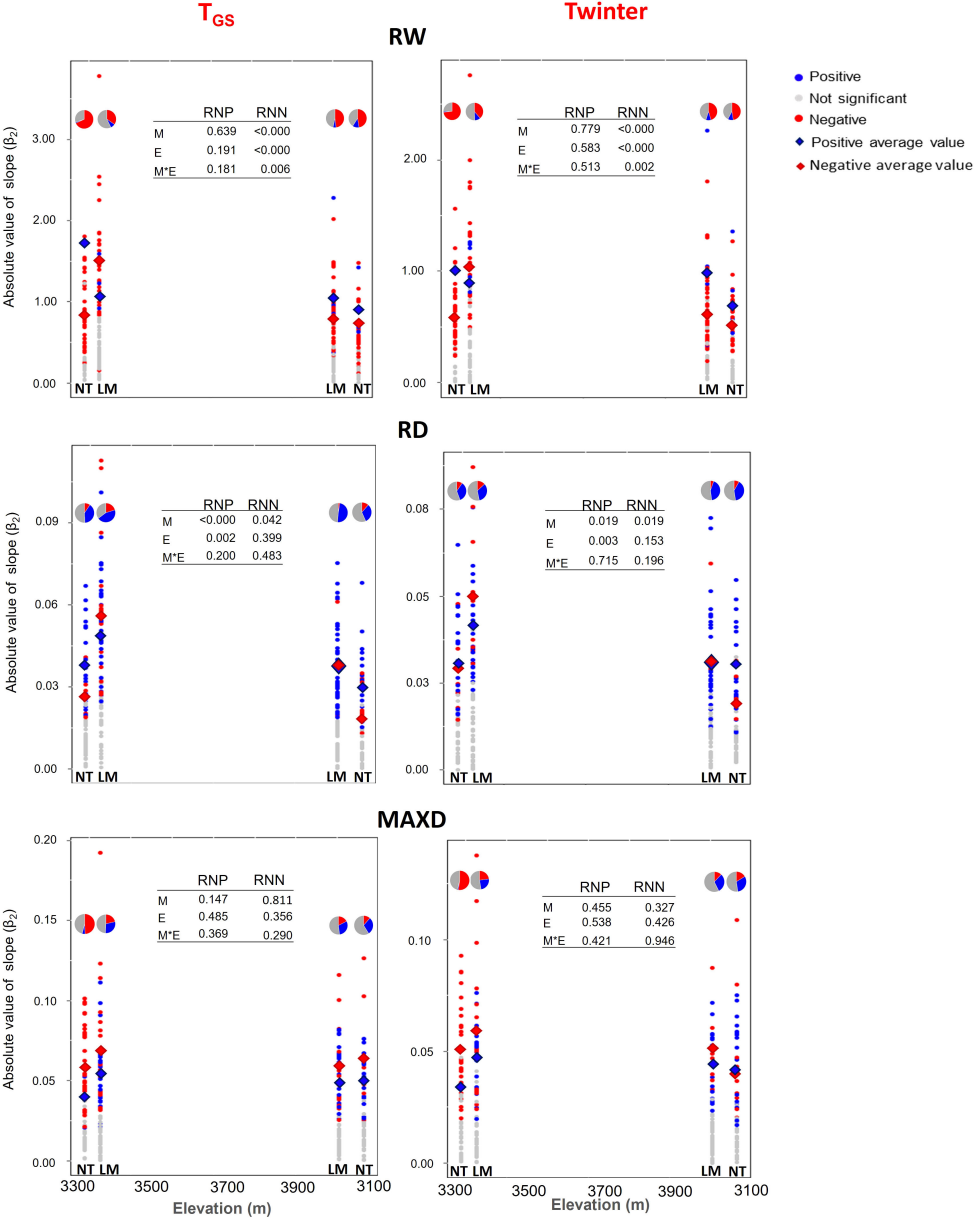
Absolute value of the slope of positive and negative RN of growth ring traits to T_GS_ (growing season temperature) and Twinter (mean winter temperature) at the lower and upper ends of the elevation gradient of *P. hartwegii* on La Malinche (LM) and Nevado de Toluca (NT) mountains. In the graph for each ring trait, we include the average value of phenotypic plasticity (PP) and the proportion of trees with positive and negative RN (pie charts). RW: Ring width (mm); RD: Ring density (g cm^-3^); MAXD: Maximum density (g cm^-3^).

A wide variation in PP was found for growth ring traits (RW, RD, and MAXD) across populations in both T_GS_ and Twinter-related NR (**Figure 5**). Even though significant RN ranged from negative to positive slopes for all traits, negative trends were more common for RW and positive trends for RD and MAXD, except for MAXD at low elevation in NT, where negative trends were predominant. In addition, there were significant elevation and mountain effects on both the proportion of trees with negative and positive RN and the mean absolute PP value for a given growth ring trait (**Figure 5**). The proportion of trees with negative trends in their RN generally decreased from the lower to the upper end for all traits in both mountains, although the effect of elevation was more pronounced for RW and MAXD at the NT mountain for both climate variables (**Figure 5**). Overall, mean absolute PP values were higher on LM than on NT for both positive (in RD) and negative (in RW and RD) RN (**Figure 5**). Also, at the lower end of the elevation gradient mean absolute PP values were higher than at the upper end for both positive (in RD) and negative (RW) RN. The effect of elevation on the absolute PP value of negative RN for RW was larger at the LM mountain (**Figure 5**). Mean absolute PP values for MAXD showed a similar trend, but differences between mountains and elevations were not significant.

## 4 Discussion

### 4.1 PP response of growth ring traits to climate variables in *P. hartwegii*

Overall, radial growth of *P. hartwegii* trees was more sensitive to temperature than to precipitation. This coincides with a previous report by Soto-Carrasco (2021) who analyzed the PP of *P. hartwegii* on three other mountains in Mexico (Mount Tláloc, Pico de Orizaba, and Cofre de Perote). In that study, precipitation was the climate variable with the fewest significant RN for growth ring traits. Liu et al. (2015) mentioned that high-mountain trees generally have low sensitivity to precipitation, although they did not propose any explanation for this finding. Hendrickson et al. (2004) and Storkey (2004) mention that, under high mountain conditions, temperature is the main factor influencing most physiological processes and phenotypic changes in plants; precipitation seems to be a limiting factor only for some processes associated with xylogenesis, particularly during extreme drought events (Pompa-García and Venegas-González, 2016; Camarero and Gutiérrez, 2017; Correa-Díaz et al., 2020; Pompa-García et al., 2021).

In our study, the differences in tree sensitivity to climatic variables could be also due to differences in the degree of micro-spatial variation in temperature versus precipitation at the study locations. Ambient temperature is more homogeneous within a single location, associated mainly with site elevation, such that the value recorded at the locality level better reflects the growing conditions of all trees on it, while rainfall (and soil moisture) is very heterogeneous over short distances (Gómez-Plaza et al., 2000; Wundram et al., 2010; Yang et al., 2018). Therefore, total annual precipitation values recorded at the site level do not necessarily reflect the moisture conditions for each tree, potentially making it more difficult to detect significant RN for them. It is worth mentioning that, although our analyses do not consider the possibility of analyzing multi-annual or lagged effects, it would be interesting to do so in future research and thus learn how individual trees within a population differ in the reaction norms of ring growth traits in response to interannual fluctuation of climatic variables, especially precipitation.

On the other hand, the sensitivity of ring growth traits to the inter-annual climate fluctuations also shows differences between mountains. There was a higher overall proportion of significant RN on NT than on LM (**Table 2**). The difference between mountains could be due to differences in the range or variation coefficient of climate variables, differences in age of trees, or to other site-level factors affecting the response of radial growth to fluctuation of climate variables. Although the variation coefficient of TAP was similar between the two mountains (14%), the average value of TAP was higher on NT and there was a larger difference between the lower and higher elevations (1458.3 mm and 1299.1 mm, respectively, compared to 1132.1 mm at the lower end and 1208.2 mm at the upper end of the elevation gradient on LM). On NT there was also a higher variation coefficient of MAT than on LM, which could have facilitated the detection of the PP response of trees in this mountain. In addition, trees sampled at NT were older, on average, than at LM (**Table 1**), which could suggest that sensitivity of radial growth to climate fluctuation increases with age in *P. hartwegii* trees. Similar findings have been reported in other studies, particularly for high mountain species (Voelker, 2011; Dominik et al., 2019). Besides, the proportion of trees with significant RN was similar at both ends of the altitudinal range, despite the broader inter-annual climate fluctuation at high elevation. At the upper end of the gradient, there was a higher variation coefficient in MAT and TAP, than at the lower end. However, environmental conditions at the upper end of the gradient are far more restrictive for vegetative growth (sub-optimal temperature and lower depth and moisture retention capacity of soil), limiting the response of trees to fluctuations in temperature and precipitation (Holtmeier and Broll, 2005; Silva et al., 2016). Despite the results obtained, we suggest that for further research, the variation of conditions within a year or the global variation between years should be considered, to corroborate that phenotypic plasticity does not refer only to the response to average climatic conditions from one year to another.

### 4.2 Relationships between the PP of growth ring traits in *P. hartwegii*

The similar correlation matrix and separation of growth ring traits in three distinctive groups, based on the PP response to T_GS_ and Twinter, shows a strong relationship between the temperature variables at different periods of the year and their effect on the growth ring traits. In other words, the PP values for any growth ring trait in response to T_GS_ and with Twinter were strongly correlated. However, the separation of ring width traits from ring density traits, and these from maximum density indicates a different plastic response, globally, of these traits to the temperature variables. The positive loadings of PC2 to RD and EWD, and negative loadings to RW and LWW show contrasting RN in these traits to increasing temperature; a positive response for the first group and a negative one for the second. MAXD also showed, globally, a negative response to increasing temperature, more pronounced in the RN to Twinter. This contrasting response in RN for width vs. density seems to indicate a compensatory effect in resource allocation at the global (population) level in response to increasing temperature, reducing cell division and size, and increasing cell wall deposition (Rozenberg et al., 2020). A reduction in the rates of cell division and/or the diameter of the cells (less ring width) and an increase in the thickness of the cell wall (higher wood density) with increasing temperature, could allow an increase in the functionality and hydraulic security of the xylem in response to increased water stress (Rozenberg et al., 2020). Several authors have pointed out that during episodes of drought or increased temperature, evapotranspiration increases, and with it, the water demand of the trees, increasing the vulnerability of the xylem to hydraulic failures due to cavitation and embolism (Bréda et al., 2006; Meinzer et al., 2010; McDowell et al., 2011).

On the other hand, the correlation analysis also indicated a low and generally non-significant correlation between the PP values for width and density traits. Thus, despite the potential compensatory effect between growth ring traits in response to temperature described at the population level, at the tree level this compensatory effect between growth ring traits was not shown. This is corroborated by the broad within-population variation in PP values found in all growth ring traits in the *P. hartwegii* populations sampled. Thus, trees differed in their PP response; they seem to have a predominant response in one or few growth ring traits, and for those with a simultaneous significant RN for several growth ring traits, the PP response was relatively independent for width and density traits. Soto-Carrasco (2021), found similar results, arguing that trees exposed to similar variations in temperature can respond differently in the PP of their radial growth traits. Similarly, Pélabon et al. (2013), indicate that high intra-population variation is common and reflects variation in the specific physiological processes associated with the phenotypic traits involved. Escobar-Sandoval et al. (2021), mention that variation in the direction of the slope of the RN among trees within the same population is possible because different trees are positioned at different points along the response curve to the climate variable, i.e., they inherently have different RN. In addition, it has been reported that the RN of the majority of phenotypic traits analyzed in plants over wide temperature intervals are generally expected to be curvilinear (Arnold et al., 2019). As such, the detection of individual trees whose significant RN have signs opposite to the general trend could suggest differences among the trees in their position on the temperature curve. However, the results could also be due to the effect of other micro-environmental factors intrinsic to each population (e.g., micro-spatial variation in temperature or soil moisture), as has been described by other authors (Diamond and Kingsolver, 2012; Tammaru and Teder, 2012; Smoliński et al., 2020). Natural tree populations are characterized by high phenotypic variation in adaptive traits, due to their inherently high genetic variation and high pollen dispersal capacity, which enables them to cope with environmental changes. If phenotypic plasticity has any adaptive role, it would also be expected to be highly variable; thus, genetic variation in climatic sensitivity for radial tree growth is also a possible cause of the variation in PP within populations at both mountains (Rolland et al., 1999; Lebourgeois et al., 2010).

### 4.3 Effect of elevation and mountain on the expression of PP in growth ring traits

The general trend of higher frequency of negative RN for ring width traits and positive RN for ring density traits was most noted at the upper end of the elevation gradient on both mountains. This partially coincides with the results of Soto-Carrasco (2021), who mentions that on the mountain Pico de Orizaba there was a higher proportion of positive RN for ring density traits at higher elevation. However, in her study, the percentage of positive and negative RN for the ring width traits did not differ between elevations. We also found several differences in the average PP values between the ends of the elevation gradient sampled. The highest values of PP for both positive and negative RN were found at the lower elevation on both mountains, for most growth ring traits in response to both T_GS_ and Twinter. These results coincide with the report by Escobar-Sandoval et al. (2021), where the absolute value of the mean slope of positive and negative RN were generally higher at the lower end of the gradient of the three mountains analyzed in that study, which was attributed to the differences in the mean temperature between the two extremes of the elevation gradient. In our study, the average value of T_GS_ during the study period was 2.0°C higher at the lower than at the upper end of the elevation gradient on LM and 4.3°C higher on NT. The difference in Twinter was even more marked, with 5.3°C and 4.5°C higher temperatures at the lower than at the higher elevation on LM and NT, respectively. A change in the slope of the RN (i.e., a change in the phenotypic plasticity) associated with a change in the range of T_GS_ and Twinter values implies that the RN associated with temperature are not linear (Arnold et al., 2019). This is the case in many biological systems (Camarero and Gutiérrez, 2004; Körner, 2012). It is common, for example, that as temperature increases, the rate of change (slope of the regression line) of metabolic processes is modified (Arnold et al., 2019; Escobar-Sandoval et al., 2021). It is therefore possible that the higher phenotypic plasticity observed at the lower end of the gradient is due to the average position of the population with respect to a non-linear RN of these traits to T_GS_ and Twinter, as has been pointed out in other studies with different growth traits in several plant species (Valladares et al., 2006; Arnold et al., 2019).

The elevational differences in PP were generally more pronounced on LM than on NT, associated in most cases with the higher average PP value at the lower end of LM (**Figure 5**). A steeper slope of the negative RN for ring width and the positive RN for ring density indicates that trees on LM are adjusting more rapidly their radial growth traits to the temperature increase. Gilmore (1968), mentions that the factors that favor a reduction in ring width generally increase their density, mainly in the second half of the radial growth period (Büntgen et al., 2010; Bouriaud et al., 2015). The differences between mountains, therefore, suggest different needs and/or responses of trees, as a function of the micro-environmental conditions on them. Even though temperature at the lower end was similar on both mountains, LM has lower annual precipitation, particularly during the summer and early fall (**Figure 1**), so trees might be experiencing higher water stress. This differential response is also shown in the wide intra-population variation in the sign and magnitude of the RN slopes found in all growth ring traits, with individuals that differed from the general population trends, discussed before. Although it is difficult to identify the main factors involved in this PP variation, the importance of PP variation as a buffering mechanism of the impact of climate change at the population level is evident, as has been mentioned by Escobar-Sandoval et al. (2021).

Therefore, *P. hartwegii* trees seem to have the capacity to show plastic responses with different growth ring traits under climate change-driven environmental variation. Populations at the lower extreme of the elevation gradient showed higher proportion of trees with negative trends in their RN to temperature and higher absolute values of PP for growth ring traits, so they seem to be experiencing higher impacts from global warming and climate change. However, the wide intrapopulation variation detected in PP of these traits offers positive perspectives on the short-term response capacity of these populations, as PP may serve as a mechanism to buffer the impact of interannual fluctuation and the gradual increase in temperature in the medium term. However, it is fundamental to obtain additional information with respect to the physiological mechanisms associated with this variation in PP, its possible genetic origin (Zacharias et al., 2022) and the adaptive implications (DeWitt and Scheiner, 2004; Ghalambor et al., 2007; Chown et al., 2008).

## 5 Conclusions

In this work we identified that ring width and density traits of *P. hartwegii* were more sensitive to temperature than to precipitation, which may be attributed to the lower variability or higher spatial homogeneity of ambient temperature at the site level. Despite the most restrictive environmental conditions at higher elevations, radial growth of trees was similarly sensitive to inter-annual climate fluctuation at both extremes of the elevational distribution gradient. Plastic response of ring traits to interannual fluctuation in temperature at different times during the year was strongly interrelated, with a clear separation of ring width from ring density traits. At the population level, a general trend was distinguished, with a negative response of ring width traits and a positive response of ring density traits to increasing temperature. At the individual level, however, plastic responses for width and density traits were mostly independent from each other, and a wide variation was found for both the sign of the RN and magnitude of PP. Both the proportion of trees with positive and negative RN to temperature and the absolute PP value differed between elevations and mountains. At the lower end of the gradient on both mountains there was a higher proportion of negative trends and higher PP values for ring width and density traits, attributed to a higher mean temperature at this elevation level and a possible non-linear response to temperature. Overall, our data show the presence of differentiated adaptive mechanisms between ring width and density traits in the PP response to temperature, with compensatory effects at the population level, but not necessarily the individual level. Apparently, *P. hartwegii* has the potential to present differential plastic responses between its ring width and density traits, with a higher degree of average PP at the lower end of the elevation gradient, associated with higher temperature and water stress. These plastic responses might be important components in the reaction of populations located at the elevational extremes of the species distribution range to the continuing increase and interannual fluctuation in temperature linked to climate change.

## Data availability statement

The raw data supporting the conclusions of this article will be made available by the authors, without undue reservation.

## Author contributions

LC-A as first author, she carried out the field sampling, laboratory analyses, as well as their description, abstract, introduction, methods, results, discussion and conclusions. JV-H was mainly involved in the statistical analysis of the work as well as in the writing of the results and the discussion of the work. This author also contributed to the field work. PR was kind enough to develop a methodology to carry out the reaction norms in this study, from the determination of the ring variables to the calculation of the plasticity value through different functions in the R software. AM-C was a guide to determine different methods to carry out the statistical analyses, and also reviewed the writing and congruence of the work in general. MP-S, as corresponding author, she was the guide for the writing of the sections such as abstract, introduction, materials and methods, as well as the discussion of the work. All authors contributed to the article and approved the submitted version.
